# Supplementary material for: Homology sensing via non-linear amplification of sequence-dependent pausing by RecQ helicase
Source: eLife. 2019 Aug 29;8:e45909. doi: 10.7554/eLife.45909 (PMC6773442; doi:10.7554/eLife.45909)
Supplement: Supplementary file 1. — Table S1: Sequence (5' to 3') of ssDNA strands composing gc36, gc46 and gc79 forked dsDNA substrates (complementary regions are bold; Flu represents 3’ fluorescein labeling). Table S2: Parameters determined from global fitting ensemble unwinding data with the n-step and delayed release models a Parameters determined from fitting ensemble unwinding data with the n-step model with n = 5 (cf. Figure 4B). b Parameters determined from fitting ensemble unwinding data with the delayed release model with n = 5 for RecQWT and n = 4 for RecQ-dH (cf. Figure 4B). Table S3 Parameters from fitting of Poff, τoff, and v as a function of ATP with three schemes. Table S4 Number of events in the analysis a: Number of events (Na+ mM), b: Number of events (ATPγS µM), c: Number of events (ATP µM) KMka [file elife-45909-supp1.docx]

**Supplementary Tables**

**Table S1: Sequence (5’ to 3’) of ssDNA strands composing gc36, gc46 and gc79 forked dsDNA substrates**

(complementary regions are bold; Flu represents 3’ fluorescein labeling)

| **gc36t** | **GAGCTGATGACTATACTACATTAGAATTCAGAG**TTTTTTTTTTTTTTTTTTTTT |
| --- | --- |
| **gc36c** | TTTTTTTTTTTTTTTTTTTTT**CTCTGAATTCTAATGTAGTATAGTCATCAGCTC –** Flu |
| **gc46t** | **CGTTGCGCAATTAAGCTCTAAGCCATCCGCAAA**TTTTTTTTTTTTTTTTTTTTT |
| **gc46c** | TCCTTTTGATAAGAGGTCAT**TTTTGCGGATGGTTAGAGCTTAATTGCGCAACG –** Flu |
| **gc79t** | **GCAGGGCGGACGGCGAGGAGGGCGCGAGCAGAG**TTTTTTTTTTTTTTTTTT TTT |
| **gc79c** | TTTTTTTTTTTTTTTTTTTTT**CTCTGCTCGCGCCCTCCTCGCCGTCCGCCCTGC –**Flu |

**Table S2: Parameters determined from global fitting ensemble unwinding data with the *n*-step and delayed release models**

|  | Description | RecQ^WT^ | RecQ-dH |
| --- | --- | --- | --- |
| *N*-step model^a^ | | | |
| *f*_P_ | Fraction of initially DNA bound helicases performing a successful unwinding run (%) (gc36, gc46, gc79) | 62 ± 3, 42 ± 2, 41 ± 5 | 79 ± 8.0, 56 ± 5.6, 28 ± 5.9 |
| *k* | Range of determined kinetic step rate constants (s^-1^) (gc36 - gc79) | 5.2 ± 0.9 – 6.3 ± 1.0 | 7.9 ± 2.2 – 9.8 ± 1.8 |
| *k*_u_ | Range of average unwinding rates (*k*_i_ * *n*; bp/s) (gc36 - gc79) | 26.1 ± 4.5 – 31.5 ± 5.1 | 39.5 ± 11 – 49.0 ± 9 |
| *k*_rebind_ | Rebinding rate (s^-1^) (gc36, gc46, gc79) | 0.0121 ± 0.003, 0.0126 ± 0.003, 0.018 ± 0.004 | 0.045 ± 0.006, 0.058 ± 0.005, 0.068 ± 0.006 |
| Delayed release model^b^ | | | |
| *A*_RecQ_ | Prefactor in Eq. S2. | 318 | 793 |
| *f*_P_ | Fraction of initially DNA-bound helicases performing a successful unwinding run (%) (gc36, gc46, gc79) | 55 ± 5, 42 ± 1, 44 ± 3 | 62 ± 6, 55 ± 5, 30 ± 5 |
| *k*_i_ | Range of determined kinetic step rate constants (s-1) | 1.69 ± 0.3 – 10.7 ± 1.8 | 5.3 ± 0.6 – 29.2 ± 3.2 |
| *k*_u_ | Range of average unwinding rates (*k*_i_ * *n*; bp/s) | 8.45 ± 1.5 – 53.5 ± 9.2 | 21.3 ± 2.4 – 116 ± 13 |
| *k*_rebind_ | Rebinding rate (s^-1^) (gc36, gc46, gc79) | 0.012 ± 0.003, 0.013 ± 0.003, 0.018 ± 0.004 | 0.093 ± 0.008, 0.059 ± 0.006, 0.087 ± 0.008 |

**^a^** Parameters determined from fitting ensemble unwinding data with the *n*-step model with *n* = 5 (cf. Fig 4B).

**^b^** Parameters determined from fitting ensemble unwinding data with the delayed release model with *n* = 5 for RecQ^WT^ and *n* = 4 for RecQ-dH (cf. Figs. 4B).

**Table S3**

**Parameters from fitting of *P*_off_ , τ_off_, and *v* as a function of ATP with three schemes**

|  | Description | A | B | C |
| --- | --- | --- | --- | --- |
| *k*_a_ | ATP binding rate (s^-1^µM^-1^); fixed | 2 | 2 | 2 |
| *k*_h_ | ATP hydrolysis rate (s^-1^); fixed | 200 | 200 | 200 |
| *k*_m_ | Rate of melting one base-pair (s^-1^); fixed | 121 | 121 | 121 |
| *k*_on_ | Rate from weak to tight DNA binding state (s^-1^) | 13.8 ± 1.9 | 13.7 ± 0.9 | 12.9 ± 0.6 |
| *k*_off_ | Rate from tight to weak DNA binding state (s^-1^) | 0.61 ± 0.06 | 1.05 ± 0.06 | 5.24 ± 0.08 |
| *K*_M_ | ATP dissociation constant (µM) | 15.7 ± 1.6 | 15.6 ± 1.1 | 15.6 ± 1.7 |
| *k*_cat_ | Maximum unwinding rate of RecQ (bp/s^-1^) | 43.9 ± 0.9 | 43.9 ± 0.9 | 43.8 ± 0.9 |
| *n* | Base pairs unwound per ATP hydrolyzed | 0.82 ± 0.06 | 0.82 ± 0.06 | 0.82 ± 0.06 |
| *k*_p_ | Rate of reannealing one base-pair by RecQ (s^-1^) | 80 ± 150 | 45.8±17.3 | NA |
| *χ^2^* | Chi squared measure of global fitting errors | 82 | 604 | 9420 |

**Table S4**

**Number of events in the analysis**

| Description | Number of events |
| --- | --- |
| 174 bp DNA hairpin unwinding by RecQ^wt^ | 55 |
| 174 bp DNA hairpin unwinding by RecQ-dH | 112 |
| 584 bp DNA hairpin unwinding by RecQ-dH  at different Na^+^ concentrations (mM)^a^ | 21 (25), 29 (50), 43 (100), 59 (150), 26 (200) |
| 584 bp DNA hairpin unwinding by RecQ-dH  at different ATPγS concentrations (µM)^b^ | 25 (50), 39 (100), 10 (200), 13 (500), 19 (750) |
| 584 bp DNA hairpin unwinding by RecQ-dH  at different ATP concentrations (µM)^c^ | 11 (5), 14 (10), 10 (20), 28 (50), 27 (100), 37 (250), 8 (500) |
| Kinetic modeling simulation | 100-300 per each case |
| The ensemble ATPγS hydrolysis by RecQ | 3 |
| 174 bp DNA hairpin with 1 mismatches | 31 |
| 174 bp DNA hairpin with 2 mismatches | 16 |
| 174 bp DNA hairpin with 3 mismatches | 11 |

^a^: Number of events (Na^+^ mM), ^b^: Number of events (ATPγS µM), ^c^: Number of events (ATP µM)
